# Methods of cognitive function investigation in the Longitudinal Study on Adult Health (ELSA-Brasil)

**DOI:** 10.1590/1516-3180.2014.1323646

**Published:** 2014-04-28

**Authors:** Valéria Maria de Azeredo Passos, Paulo Caramelli, Isabella Benseñor, Luana Giatti, Sandhi Maria Barreto

**Affiliations:** I MD, PhD. Associate Professor, Department of Internal Medicine, School of Medicine, Universidade Federal de Minas Gerais (UFMG), Belo Horizonte, Minas Gerais, Brazil; II MD, PhD. Full Professor, Department of Internal Medicine, School of Medicine, Universidade Federal de Minas Gerais (UFMG), Belo Horizonte, Minas Gerais, Brazil; III MD, PhD. Associate Professor, Department of Internal Medicine, School of Medicine, Universidade de São Paulo (USP), São Paulo, Brazil; IV MD, PhD. Adjunct Professor, School of Nutrition, Universidade Federal de Ouro Preto (UFOP), Ouro Preto, Minas Gerais, Brazil; V MD, PhD. Full Professor, Department of Preventive and Social Medicine, School of Medicine, Universidade Federal de Minas Gerais (UFMG), Belo Horizonte, Minas Gerais, Brazil

**Keywords:** Cognition, Prevalence, Incidence, Prognosis, Adult, Cognição, Prevalência, Incidência, Prognóstico, Adulto

## Abstract

**CONTEXT AND OBJECTIVE:**

Many uncertainties concerning risk factors and evolution of cognitive disorders remain. We describe the methods and preliminary results from the investigation of the cognitive function in the Longitudinal Study on Adult Health (ELSA-Brasil).

**DESIGN AND SETTING:**

Multicenter cohort study on public employees at six public teaching and research institutions.

**METHODS:**

The participants were interviewed and examined to obtain a broad range of social, clinical and environmental characteristics. The following standardized tools were used to assess memory, language and visuospatial and executive functions: words or figure memory test; semantic (animals) and phonemic (letter F) verbal fluency tests; and trail test B.

**RESULTS:**

15,101 out of 15,105 participants took the cognitive tests: 54% were women; the mean age was 51 years; and 52% had a university degree. 14,965 participants (99%) did the word test and 136 (1%) did the figure test due to low schooling level. The scores from the semantic verbal fluency tests (mean = 18.42 ± 5.29; median = 18 words) were greater than the scores from the phonemic verbal fluency tests (mean = 12.46 ± 4.5; median = 12 words). The median time taken to perform the trail test was 1.6 minutes.

**CONCLUSION:**

The large cohort size, of young age, and the extensive amount of clinical and epidemiological data available will make it possible to investigate the prognostic value of biological, behavioral, environmental, occupational and psychosocial variables over the short and medium terms in relation to cognitive decline, among adults and elderly people.

## Abstract (pt)

**CONTEXTO E OBJETIVO:**

Ainda persistem muitas incertezas relativas a fatores de risco e evolução das desordens cognitivas. Descrevemos métodos e resultados preliminares da investigação da cognição no ELSA-Brasil, Estudo Longitudinal de Saúde do Adulto.

**TIPO DE ESTUDO E LOCAL:**

Estudo de coorte, multicêntrico, com servidores públicos de seis instituições públicas de ensino e pesquisa.

**MÉTODOS:**

Participantes foram entrevistados e examinados, para obtenção de amplo espectro de variáveis sociais, clínicas e ambientais. Foram utilizados instrumentos padronizados de avaliação da memória, linguagem e funções executiva e visual espacial: teste de memória de palavras ou de figuras, de fluência verbal semântica (animais) e fonêmica (letra F) e teste de trilhas B.

**RESULTADOS:**

15,101 de 15,105 participantes fizeram os testes cognitivos; 54% eram mulheres, a média da idade foi de 51 anos e 52% tinham grau universitário. 14.965 participantes (99%) fizeram o teste de palavras e 136 (1%) o teste de figuras, dado o menor grau de instrução. Os escores dos testes de fluência verbal semântica (média = 18.42 ± 5.29, mediana = 18 palavras) foram maiores que os escores dos testes de fluência verbal fonêmica (média = 12.46 ± 4.5, mediana = 12 palavras). O tempo mediano para execução do teste de trilhas foi de 1,6 minutos.

**CONCLUSÃO:**

O grande tamanho da coorte, de idade jovem, e a extensa quantidade de dados clínicos e epidemiológicos disponíveis permitirão a investigação do prognóstico de variáveis de natureza biológica, comportamental, ambiental, ocupacional e psicossociais, em curto e médio prazo, sobre o declínio cognitivo em adultos e idosos.

## INTRODUCTION

The Longitudinal Study on Adult Health (ELSA-Brasil) is a cohort of public employees at six public teaching and research institutions in Brazil: Federal University of Bahia (Universidade Federal da Bahia, UFBA), Federal University of Espírito Santo (Universidade Federal do Espírito Santo, UFES), Federal University of Minas Gerais (Universidade Federal de Minas Gerais, UFMG), University of São Paulo (Universidade de São Paulo, USP) and Oswaldo Cruz Foundation (Fundação Oswaldo Cruz, Fiocruz). This study is investigating the incidence and evolution of chronic diseases, especially cardiovascular diseases and diabetes, among adults.^1^ In this article, we present the methodology of the cognitive function investigation and the sociodemographic characteristics of the study population.

The increases in life expectancy and the rapid aging process observed in developing countries are occurring in a context of social inequalities and structural difficulties, since these countries are institutionally unprepared to meet the great demands associated with ageing. Even though aging is a victory for humanity, it increases the risk of chronic diseases and functional dependency. Over the next few years, an alarming number of Brazilians will reach an age at which the incidence of dementia is high. Estimates from population studies in Latin America and Brazil have shown that the prevalence of dementia increases from less than 10% up to the age of 75 years to more than 40% among individuals aged 85 years or over, which is precisely the age stratum that is expanding most rapidly among elderly Brazilians.^2^

Dementia is a serious syndrome that compromises the memory and at least one more cognitive function, thereby leading to declining social and occupational capacities. Moreover, cognitive impairment without dementia has emerged as an important clinical entity, and its prevalence is twice as high as the prevalence of all forms of dementia combined. In addition, it has high rates of progression towards dementia, estimated to be 10% to 15% a year, in relation to the rates among individuals without cognitive deficit (1% to 2% a year).^3^ The question that therefore arises is whether memory loss without dementia is a benign process, with slow progression; or whether it should be understood as the initial phase of more significant cognitive losses, which could lead to dementia. A five-year follow-up study found that people with cognitive impairment, but without dementia presented higher risks of diagnoses of dementia (47% versus 15%), or dying (49% versus 30%), or being admitted to long-term institutional care (29% versus 14%), in comparison with those without cognitive impairment.^4^

There is evidence that the decline in cognitive function begins during adult ages, even before the age of 50 years, and that its development is continuous over a 20 to 30-year period.^4^ However, not all components of cognitive function are equally affected by age: environmental, social and behavioral factors are also determinants in this process. Nonetheless, there are still many doubts regarding the role of risk factors, and the quality and extent of these changes. There are few cohort studies on cognitive disorders in Latin America; most of them are concentrated on the elderly population.^2^ ^,^ ^5^ ^,^ ^6^ Developed countries have already a significant number of community-based cohort studies on adults in the age range of 45-64 years, i.e. "middle-age", although most studies are still concentrated on the elderly.^7^ ^-^ ^9^

Many studies have suggested that cardiovascular risk factors contribute towards development of dementia, and cohort studies have demonstrated a consistent association between midlife health situations and the incidence of late-life dementia. Midlife hypertension presented a more significant association with the incidence of dementia than does late-life hypertension.^10^ ^-^ ^12^ Treatment for hypertension has been correlated with lower incidence of dementia, but the results are still difficult to interpret.^13^ ^,^ ^14^ Midlife diabetes has been correlated with late-life dementia in seven cohort studies (relative risk, RR: 1.2-1.7), and it persists as a risk factor even during later life. This correlation is higher in long-term diabetes cases.^15^ Hyperinsulinemia has been significantly correlated with lower cognitive tests scores in cross-sectional data and, after a six-year follow-up, in adults aged from 45 to 65 years.^16^ Midlife obesity has also been associated with a higher risk of dementia, while doing physical activity seems to contribute to lower risks of mild cognitive impairment, Alzheimer's disease and any type of dementia*.* ^17^ ^,^ ^18^ Current smoking, but not former smoking, has been correlated with higher risks of having Alzheimer's disease and also with other causes of dementia.^19^ Ingestion of small quantities of alcohol can be protective against dementia and Alzheimer's disease, but not against vascular dementia.^20^

There is also evidence that presence of psychosocial factors of an intellectually stimulating nature throughout life can contribute towards increased cognitive reserve and thus prevent, attenuate or delay dementia. These factors include schooling level, type of occupation, social capital and minor mental disorders. In a study among Afro-Americans with low schooling levels, occurrence of adverse medical conditions, particularly stroke, had a great impact on alterations in memory and executive function in adults (51-64 years).^21^ In a cohort of British middle-aged workers, socioeconomic position during childhood did not reveal any direct effect on performance in cognitive tests, but had an indirect effect, mediated by schooling level and socioeconomic situation during adulthood.^22^ In this same cohort, it was observed that long working hours (55 hours versus 40 hours per week) negatively affected cognition.^23^

Low schooling level may be a risk factor for cognitive impairment, but it is also one of the confounders in studies that have evaluated cognition tests that have the prerequisites of the ability to read and calculate, and of vocabulary diversity, among others. Studies have demonstrated worse performance among individuals with low schooling levels in verbal fluency tests and the trail-making test.^24^ ^,^ ^25^

Common mental disorders, especially anxiety and depression, have great interaction with cognitive function. They can be risk factors for cognitive deficits, confounding factors in performing cognitive tests, or even part of the initial signs of dementia (protopathic bias).^26^

Despite extensive research, many uncertainties concerning risk factors for dementia remain. These uncertainties include lack of comparability and standardization of the outcomes (performance in tests; dementia or Alzheimer's disease); study design (cross-sectional, cohort or clinical trial); diversity of the samples included in the studies (Japanese men, nuns, white women and British workers); exposure (throughout life, midlife or late life); and diagnostic instruments (cognitive tests, neurological examinations or brain imaging). There have also been some difficulties in including the diagnoses of certain types of dementia.

In 2010, after reviewing the literature on risk factors for Alzheimer's disease, a panel of specialists took the view that strong evidence only exists regarding genetic factors (variation of the Apo E gene), and that the evidence was weak in relation to the effects of midlife hypertension, diabetes, depression, current smoking, low-fat diets, folic acid intake, moderate alcohol consumption, low social support and never having been married. They also concluded that there was no consistent association between the effects of obesity, metabolic syndrome and blood pressure and use of anti-hypertensive medications, non-steroidal anti-inflammatory drugs or gonadal steroids. They indicated that the following were the main deficiencies of epidemiological studies: difficulty in distinguishing between association and causality; lack of standardization in defining cases; and complexity in establishing whether associated variables, such as depression and weight loss, are risk factors or, in reality, are early signs of Alzheimer's disease.^27^

## OBJECTIVES

The aim here was to present the methodology used to investigate cognitive function and the sociodemographic characteristics of the study population of ELSA-Brasil.

## METHODS

The baseline data collection was completed in 2010, and all participants were interviewed and examined in order to obtain a comprehensive range of psychosocial, environmental and clinical characteristics (Table 1). Since then, telephone follow-up interviews have been taking place annually and the first three-year interval follow-up examinations were conducted in September 2012. The object of the cognitive investigation was to identify changes in cognitive tests, which may be of transitory nature and may or may not be reversible, and to identify irreversible final outcomes such as occurrences of all types of dementia. Other than the abovementioned strategies, it was planned that occurrences of outcomes would be investigated by correlating data with secondary databases such as mortality and hospitalization rates.^1^

**Table 1:** ELSA (Estudo Longitudinal de Saúde do Adulto) components of interviews, examinations and laboratory measurements that will be available for cognitive investigation1

Variables	
Sociodemographic characteristics	Age, sex, race/ethnicity, educational level, income, religion, marital history and childhood living conditions
Health and medical history	Self-rated health and medical histories of cardiovascular diseases, diabetes, cancer and other selected chronic diseases of interest; investigation of rose angina and intermittent claudication; questionnaires on heart failure and headache
Mental health	Clinical Interview Schedule Revised (CIS-R)
Occupational exposure	Job characteristics (stress, degree of autonomy, access to funds and authority) and retirement situation
Family history	Cardiovascular disease, diabetes and sudden death
Reproductive health	Menarche and menopause, contraceptive use, reproductive history and hormone therapy
Healthcare	Access to preventive healthcare/examinations, health insurance and healthcare utilization
Psychosocial factors	Neighborhood characteristics (leisure, sports and access to food purchasing), stressful life events and self-rated social status
Habits	Food frequency, smoking, alcohol consumption and current physical activity
Medication	Prescription and nonprescription drugs, vitamins, dietary supplements and other remedies taken in past month.
Physical examinations	Weight, height, blood pressure and ankle brachial index
Clinical tests	Electrocardiography, transthoracic echocardiography and carotid scan
Laboratory tests	Total blood cell count, plasma glucose, oral glucose tolerance test, HbA1c, creatinine, total, HDL and LDL-cholesterol, triglycerides, gamma-glutamyl transferase, AST and ALT, Uric acid, Na, K and Ca, microalbuminuria, high-sensitivity C-reactive protein, thyroid-stimulating hormone, thyroxine, insulin and Chagas disease serology

HDL = High density lipoproteins

LDL = Low density lipoproteins

AST = Aspartate transaminase

ALT = Alanine transaminase

Every public employee at the institutions involved, between the ages of 35 and 74 years, was considered eligible. Individuals who intended to leave the institution, those who presented severe cognitive deficit, pregnant women, those who were retired and those who did not live in one of the six metropolitan regions were excluded. The sample size was calculated based on the incidence of type 2 diabetes and myocardial infarction and resulted in 6,400 participants. The cohort was composed by volunteers and a random sample was derived from it. ELSA has a dual system of recruitment, in which part of the sample consists of volunteers and part is derived from an actively recruited random sample of eligible individuals drawn from lists from the institutions.^1^ The total sample would be used to examine the association of risk factors with cognitive decline and dementia, while the random sample would make it possible to estimate the frequency and distribution of cognitive deficits.

Table 2 shows the psychometric characteristics of the neuropsychological tests. The choice of these tests was based on the following criteria: 1) evaluation of multiple cognitive domains: memory (word learning, retention and recognition tests) and executive functions (verbal fluency tests: semantic (animals) and phonemic (first letter); and trail-making test version B; 2) national and international comparability; 3) adaptation to Portuguese; 4) normative rules for diagnosing cognitive deficits; and 5) compatibility between the time of cognitive test application and the time reserved for the whole ELSA-Brasil questionnaire. A "Standardization Manual for Application of Cognitive Tests" was drawn up to ensure and control the quality of the study.

**Table 2:** Description of the cognitive tests used in ELSA-Brasil study (Estudo Longitudinal de Saúde do Adulto)

Test	Main domain	Domain capacities
CERAD word test* (Figure test†)	Memory	Immediate learning of new information; retention of information after three trials
Word recall (Figure recall)		Delayed recall of the learned information
Word recognition(Figure recognition)		Remembering and distinguishing the correct words from a set of distractors
Verbal fluency tests (animals and letter F)	Executive functions	_ Language (also semantic memory) _ Attention to a task over time _ Concentration _ Use of information learned in the test instructions _ Visual-spatial organization; connecting words and numbers _ Speed (of speaking correct words or connecting words and numbers) _ Cognitive flexibility: recognition and correction of possible errors
Trail-making test (version B)		

* CERAD words, Brazilian version: poste, motor, carta, braço, manteiga, erva, cabana, árvore, bilhete, praia. ^28^

† Figure test images: shoe, house, comb, airplane, key, bucket, turtle, book, spoon, tree. ^29^

The Consortium to Establish a Registry for Alzheimer's disease (CERAD) has been adapted for Brazilian studies. It includes a list of ten unrelated words printed in large letters on cards, which are shown at a rate of one every two seconds and are presented in a different order in each of the three learning trials, with immediate recall. After a five-minute delay, retention is tested by means of free recall through the recognition trial, in which the ten previous words are intermixed with ten distractor words.^28^ A figure test consisting of simple drawings from the Brief Cognitive Screening Battery (BCSB), which follows the same logic as the word test was offered to participants who were unable to read.^29^ The verbal fluency tests consisted of asking participants to say as many words as possible relating to a specific category of animals (semantic test) or starting with the letter F (phonemic test), in one minute. To perform the trail-making test version B, the participant was instructed to draw lines connecting letters and numbers in an order that alternated between increasing numeric value and alphabetic order (1, A, 2, B, 3, C etc.). The participant was told to draw as quickly as possible, without lifting the pencil point from the page. The supervisors were instructed to point out the errors. The test score was the total time taken to complete the task, including the time need to correct errors.^28^

The training and certification for the interviewers were standardized and centralized, and were provided by the same researcher (VMA Passos). During the training, each interviewer applied a battery of tests to at least three volunteers, who had the same characteristics as the participants regarding sex, age and schooling level. The level of achievement and the basic items used to evaluate each interviewer's performance were discussed after each interview in order to reach an agreement among the central and local supervisors. This strategy allowed the local supervisors to implement subsequent training, in the event of substitution of members of the team. The certification consisted of a theory test, on topics from the application manual, and a practical test that entailed applying an interview to a volunteer. This process was also followed by each local supervisor, who had been trained to certify new interviewers in the event of alterations to the team during the field work.

In a test and retest study with a mean interval of 29 days, which was conducted on 160 participants, higher scores were observed in all the retests, as well as a decrease in the average time taken to do the trail-making test version B. The memory tests presented moderate reliability, the verbal fluency tests presented good reliability and the trail-making test version B presented almost perfect reliability.^30^

The verbal fluency tests require that interviewer should write down all the words said by the participant during a one-minute period. To avoid mistakes in the transcriptions, they were all recorded and checked. The supervisor of each center was also trained to calculate verbal fluency test scores, and a reliability study was designed to ascertain the degree of homogeneity of the interpretation among the ELSA centers. Intraclass correlation coefficients and Bland-Altman plots were used to examine patterns of rating disagreement among the verbal fluency test scores given by the supervisors at the six ELSA research centers, who independently judged 120 category tests (animals) and 120 phoneme tests (letter F). Their scores were compared with a reference standard score that was obtained through independent judgment by two experts. The scores were very similar among the ELSA centers, and a high level of agreement was observed between each center and the reference standard.^31^

The data center for delineating and managing the data system was operated by a multidisciplinary team. A web-based system was developed, in order to enable online data entry, verification and editing while maintaining security and confidentiality, as well as in order to incorporate data that had been gathered on paper and obtained from reading centers. Processes for data extraction and cleaning were also developed in order to create databases in formats that would allow analyses in multiple statistical groups.

ELSA-Brasil was approved by the Research Ethics Committee of all the six participating centers and by the National Research Ethics Committee. All the participants signed a free and informed consent statement before collection of any clinical and laboratory data. All international rules regarding data confidentiality during storage and analysis are being followed.^1^

## RESULTS

The vast majority of the participants (15,101 out of 15,105) took the cognitive tests; 54% of them were women and 46% were men. There were four cases of refusal to participate. The age range was from 35 to 74 years, with a median age of 51 years. With regard to working status, 80% were active public employees and 20% retired. Over 52% (7,498) had a university degree, 4204 (27.8%) had completed high school, 1898 (12.6%) had completed middle school, 1029 (6.8%) participants had studied in but not completed middle school and 22 (0.2%) were illiterate.

Significant differences in the distribution of sociodemographic characteristics according to investigation center were observed. There was an overall predominance of women, with the highest contingents in the states of Bahia and Rio Grande do Sul. The mean age was 52.00 ± 9.08 years, with the youngest participants in the state of Rio de Janeiro, where the number of retired participants was also lower than in the other centers. Only 10% of the participants had attended school for less than five years, and the lowest percentages were found in Rio de Janeiro and Rio Grande do Sul (Table 3).

**Table 3:** Sociodemographic characteristics of the 15,101 participants in the ELSA study (Estudo Longitudinal de Saúde do Adulto) who attended cognitive tests in baseline exami

Variables	Investigation centers						
	Bahia n (%)	Espírito Santo n (%)	Minas Gerais n (%)	Rio de Janeiro n (%)	Rio Grande do Sul n (%)	São Paulo n (%)	Total n (%)
Sex							
Male	842 (41.50)	497 (47.20)	1474 (47.32)	853 (47.81)	887 (43.08)	2336 (46.16)	6,892 (45.64)
Female	1187 (49.50)	556 (52.80)	1641 (52.68)	931 (52.19)	1172 (56.92)	2725 (53.84)	8,213 (54.36)
	χ2 = 27.99; P < 0.0001						
Age							
Mean	53.62	52.79	52.42	49.52	53.12	51.61	52.09
Standard deviation	9.17	8.86	8.97	8.12	9.58	9.05	9.08
	F = 51.08; P < 0.0001						
Schooling							
1-4 years	298 (14.69)	159 (15.10)	296 (9.50)	47 (2.63)	39 (1.89)	801 (15.82)	1640 (10.86)
≥ 5 years	1731 (85.31)	894 (84.90)	2819 (80.50)	1737 (97.37)	1740 (98.11)	4260 (84.18)	13465 (89.14)
	χ2 = 26.33; P < 0.0001						
Working status							
Employee	1560 (76.89)	828 (78.63)	2466 (79.16)	1626 (91.14)	1487 (72.22)	4129 (81.58)	12,097 (80.10)
Retired	469 (23.11)	225 (21.17)	649 (20.84)	158 (9.86)	572 (27.78)	932 (18.42)	3,008 (19.90)
	χ2 = 240.00; P < 0.0001						

14,965 participants (99%) completed the three learning trials of the word memory test, and only 136 (1%) had to be given the figure memory test because of poor literacy skills. In both tests, the participants' scores increased at the second and third attempts with means of 4 ± 1.6, 7.4 ± 1.6 and 8.2 ± 1.4 for the first, second and third attempts at the word memory test and 5.7 ± 2.1, 7.2 ± 2.4 and 7.3 ± 2.4 for the figure memory test.

The scores for the semantic verbal fluency tests (mean = 18.42 ± 5.29 words; median = 18 words) were greater than the scores for the phonemic verbal fluency test (mean = 12.46 ± 4.5 words; median = 12 words).

The overall time taken to perform the trail-making test version B, without excluding outliers, ranged from 0.2 minutes to 26 minutes (mean = 2.1 ± 1.45 minutes; median = 1.6 minutes).

## DISCUSSION

Transcultural studies have already identified significant variability in how dementia is expressed in different populations.^12^ Establishment of a cohort of public employees in six Brazilian states, including adults and elderly people, will make it possible to investigate the influence of various social and clinical characteristics of the participants on their cognition, from middle age onwards.

Investigation of cognitive impairment at the baseline will make it possible to evaluate the prevalence and factors associated with cognitive impairment among the adult population by comparing the test results from different age strata, with adjustments for the different intervenient variables investigated. This first approach has the merit of providing an understanding of the distribution of cognitive functions in the study population, but presents the disadvantages inherent to this type of cross-sectional design.

The prospective analysis will enable us to compare intra-personal performance, without the birth cohort effect, thus making it possible to measure the potential influence of risk factors, such as lifestyle, presence of diseases and use of medications. In addition to investigation of the natural evolution of cognition, the longitudinal study will allow us to investigate modifiable risk factors for cognitive decline, which is a public health priority, in an effort to prevent or delay occurrences of dementia.

Cognitive tests are used as a proxy for the participants' cognition. They present an advantage over clinical evaluation, in that they establish standardized scores, thus allowing comparison over time between people and studies. However, for this comparison, it has to be assumed that the cognitive tests have stable and reproducible results, which is not always true. The high consistency of verbal fluency test scores confirms that these tests are reliable and valid, and assures their use in multicenter studies.

As observed, reapplication of these tests, particularly when the interval between applications is short, may lead to an improvement in performance, due to the learning effect. With the aim of minimizing this learning effect in the ELSA follow-up, in 2012, the ten CERAD words used in the memory test will be presented in a different order and the tests of verbal fluency will demand a different category (vegetables) and a different initial letter (A). The trail-making test version B, which presented high reproducibility when reapplied after short period, will be repeated. The baseline cognitive tests will be repeated exactly, in the second ELSA follow-up, after an interval of six years. This strategy will make it possible to investigate the influence of confounder factors on cognition, such as learning effects, on test performance.

Always bearing in mind the aphorism *Brain function is too complex to be communicated in a single score*, many other investigation strategies will be used during the development of the cohort, such as "nested case-control studies". Other tools for cognitive analysis will also be included, such as clinical-neurological evaluation and imaging tests.^32^ Multifactorial analysis will be carried out, and the reliable change index will be calculated in order to determine any real and significant changes in cognitive test scores over time.^33^

## CONCLUSION

Investigation of cognitive function decline in ELSA-Brasil takes into consideration its insidious onset, its long latency period and the need to identify factors that interact with the evolution of these processes. These approaches are only possible in longitudinal studies that include a large adult population. Such studies make it possible to identify the subclinical processes of dementia and are a source of knowledge for intervening in these risk factors, especially in modifiable ones.
